# Nutraceuticals as Ligands of PPAR**γ**

**DOI:** 10.1155/2012/858352

**Published:** 2012-06-20

**Authors:** Meera Penumetcha, Nalini Santanam

**Affiliations:** ^1^Division of Nutrition, BFLSON and Health Professions, Urban Life Building, 140 Decatur Street, Suite 862, Atlanta, GA 30303, USA; ^2^Department of Pharmacology, Physiology and Toxicology, Joan C Edwards School of Medicine, Marshall University, One John Marshall Drive, Huntington, WV 25755, USA

## Abstract

Peroxisome proliferator-activated receptors (PPARs) are ligand-activated nuclear receptors that respond to several exogenous and endogenous ligands by modulating genes related to lipid, glucose, and insulin homeostasis. PPAR*γ*, expressed in adipose tissue and liver, regulates lipid storage and glucose metabolism and is the target of type 2 diabetes drugs, thiazolidinediones (TZDs). Due to high levels of toxicity associated with the first generation TZDs, troglitazone (Rezulin), rosiglitazone (Avandia), and pioglitazone (Actos), there is a renewed search for newer PPAR drugs that exhibit better efficacy but lesser toxicity. In recent years, there has been a definite increase in the consumption of dietary supplements among diabetics, due to the possible health benefits associated with these nutraceutical components. With this impetus, investigations into alternative natural ligands of PPARs has also risen. This review highlights some of the dietary compounds (dietary lipids, isoflavones, and other flavanoids) that bind and transactivate PPAR*γ*. A better understanding of the physiological effects of this PPAR activation by nutraceuticals and the availability of high-throughput technologies should lead to the discovery of less toxic alternatives to the PPAR drugs currently on the market.

## 1. Introduction

Peroxisome proliferator-activated receptor gamma (PPAR*γ*), or NRIC3, is a ligand-activated transcription factor that belongs to the superfamily of nuclear receptors. PPAR*γ* plays an important role in glucose and lipid homeostasis, inflammation, and adipocyte differentiation [1]. There are three known isoforms of PPARs: PPAR*α*, PPAR*γ*, and PPAR*β*/*δ*, each with different tissue specificity and physiological function [2]. All three isoforms share common molecular structure and functional domains similar to other nuclear receptor superfamilies consisting of the following: a distinct N-terminal ligand-independent transcriptional activation domain (AF-1), a DNA binding domain, the hinge region, and the ligand-binding domain which contains the ligand-dependent transcriptional activation domain (AF-2). Upon ligand binding, PPAR*γ* forms a heterodimer with the retinoic acid receptor (RXR) and controls the expression of genes that have PPAR response elements (PPRE). This transcription factor is further regulated by commonly known coactivator proteins such as CBP/p300, the SRC family, TRAP 220, and corepressors such as SMART, NCoR, and RIP140 [1]. Two isoforms of PPAR*γ* have been identified (PPAR*γ*1 and PPAR*γ*2), with a wide tissue distribution among various animal species [3].

Over the past two decades, there has been a flurry of research investigating the physiological significance of PPAR*γ* activation. It is now generally accepted that both ligand dependent and independent activation of PPAR*γ* mediate multiple metabolic pathways in the immune system [4], cardiovascular system [5], and the adipose tissue [6], thus modulating genes related to inflammation, lipid metabolism and adipogenesis. Most of these physiological functions of PPAR*γ* were revealed because of the discovery of thiazolidinediones (TZD). These drugs are high affinity ligands of PPAR*γ* with insulin sensitizing effects and used in the treatment of type 2 diabetes [7]. The identification of PPAR*γ* as the molecular target of glitazones such as pioglitazone (TZD), came from seminal work by Kliewer et al. [8], Kletzien et al. [9], and Graves et al. [10]. Troglitazone (Rezulin), rosiglitazone (Avandia), and pioglitazone (Actos) were the three originally approved TZD drugs for diabetes. Increased hepatic toxicity, edema, and cardiovascular risk associated with the use of the TZD drugs lead to the withdrawal of troglitazone (Rezulin) from the market and black box warnings on the other two available drugs [11]. Although these drugs are known PPAR*γ* agonists, it is still not clear if the toxicities associated with these drugs are due to their interactions with the PPAR*γ* receptor. A new generation of PPAR*γ* drugs with equivalent insulin sensitizing activity like TZDs, but with lower toxicity, has been in development since the withdrawal of the earlier TZDs. These include (i) non-TZD like PPAR*γ* agonists, (ii) PPAR *α*/*γ* dual agonists, (iii) PPAR pan agonists, (iv) PPAR*γ* antagonists, and (iv) selective PPAR*γ* modulating drugs (SPPAR*γ*M) [12, 13]. These newer agonists seem to have similar or better insulin sensitizing effects as compared to TZDs (rosiglitazone). Still, several of these new drugs exhibit some form of toxicity [14]. Yet, SPPAR*γ*M are purported to be less toxic because they are designed based on the ligand selective regulation of receptor function [13, 15–17]. Recent studies indicate that SPPAR*γ*M are mechanistically distinct from the TZDs in that these drugs interact at a site that is different than the AF-2 region, thus altering subsequent coregulator binding and resulting in favorable cellular responses [18]. The search will continue until better alternative drugs to the currently available TZDs with equal or greater beneficial effects, but fewer adverse effects are identified. 

## 2. Natural Ligands of PPAR*γ*

Although there is a renewed interest in identification of synthetic PPAR*γ* modulators for the treatment of type 2 diabetes, developing known dietary components (nutraceuticals) that bind and activate PPAR*γ* with more efficacy and safety, while promoting health benefits has become an absolute necessity [19]. The term nutraceutical is defined as any food (fruits, vegetables, nuts, tea, etc.) or part (extract) of a food, such as a dietary supplement that has a medical or health benefit including the prevention and treatment of disease [20]. However, there is no consensus on the definition or the regulation of nutraceuticals among scientists [21]. The majority of nutraceuticals are of plant origin. Thus, nutraceuticals are “pills” that contain concentrated forms of presumed bioactive phytochemicals extracted from the original food item (e.g., genistein from soy). Because of their plant origin, these compounds are considered safe and are popular among consumers. This review will elaborate on some of the currently well-known dietary constituents that act as PPAR*γ* ligands, with a demonstrated ability to bind to and activate PPAR*γ*. The subsequent biological responses that result from this activation is not the focus of this review. For the purposes of this review, any isolated dietary component used in cell based or animal studies is considered a nutraceutical. Dietary components that act as ligands of PPAR*γ* include dietary lipids such as n-3 and n-6 fatty acids and their derivatives, isoflavones and flavonoids.  Table 1 provides a partial list of dietary PPAR*γ* ligands.

### 2.1. Exogenous and Endogenous Lipid Derivatives

The majority of available research has focused on understanding the physiological significance of the interactions between dietary lipids and their derivatives with PPARs [25, 32–38]. Dietary fats and oils are major sources of these ligands, which include both n-3 and n-6 lipids and their oxidized counterparts. Elegant structure-function studies have determined the binding efficiency of the dietary lipids with PPARs [25, 39–42] by comparing them to synthetic drugs (TZD). Though dietary lipids similar to synthetic ligands were able to bind to the ligand binding domain and cause conformational changes to activate the receptor, they are considered as weak PPAR*γ* ligands because of their low physiological concentrations. One must keep in mind that most of the studies determining the binding efficiency of the nutraceuticals have been performed in either cell-free or cell-based systems. The specificity of the dietary compounds to act as ligands for PPAR*γ* was determined by a lack of response when cells were either pretreated with a known antagonist of PPAR*γ* or with constructs that lacked PPAR ligand binding domain. However, in cell based systems it is conceivable that a metabolite of the parent compound, not the parent compound itself, might be mediating the response through interactions with PPAR*γ*. For example, 13-HODE (oxidized n-6 lipid), a known agonist of PPAR*γ*, could be converted into 13-Ox-HODE prior to interacting with PPAR*γ*.

#### 2.1.1. Exogenous Lipids: Dietary Lipids

Many studies have demonstrated that nonesterified unsaturated fatty acids are better ligands of PPAR*γ* as compared to saturated fatty acids [43]. Although unoxidized unsaturated fatty acids are present in abundance *in vivo*, evidence suggests that they are weak activators of PPAR*γ*. However, there is compelling evidence that oxidized unsaturated fatty acids are potent ligands compared to their unoxidized counterparts. Using NMR spectroscopy, Itoh and colleagues [39] studied the crystal structure of PPAR*γ* bound fatty acids. They determined that fatty acids that bound covalently to the receptor were strong activators of PPAR*γ* and the binding was also dependent on the polar nature of the lipid. Furthermore, using a dual luciferase reporter system, they demonstrated that the oxidized forms of the docosahexaenoic acid (DHA), a dietary n-3 fatty acid, 4-hydroxy docosahexaenoic acid (4-HDHA), and 4-oxo docosahexaenoic acid (4-oxoDHA) were potent ligands (EC_50_ values of 3.7 *μ*M and 0.4 *μ*M) as compared to DHA (>10 *μ*M). Fatty acids that are modified by oxidation or nitration can originate in the diet or can be generated *in vivo*. Research from our laboratory [44] and by others [45, 46], has shown that dietary oxidized lipids are absorbed by the intestine and incorporated into lipoproteins and tissues. A study by Ringseis et al. [47] showed increased PPAR*γ* DNA binding in the intestinal cells of pigs fed oxidized (heated) sunflower oil compared to pigs fed unoxidized oil. Even though it was not possible to identify the specific ligands that bound to PPAR*γ*, the findings from this study are important because they demonstrated that dietary oxidized fats were able to increase PPAR*γ* interactions with the DNA, even though this activation of PPAR*γ* was not associated with concomitant NF*κ*B mediated inflammation. Seminal work by Schopfer et al. [22] has shown that nitrolinoleic acid (LNO2), which acts as a PPAR*γ* ligand, is present in the plasma of healthy humans and has a *K*_*i*_ of 133 nM as compared to a *K*_*i*_ of >1000 nM for linoleic acid. Additionally, it was capable of promoting adipogenesis and glucose uptake in the 3T3-L1 cell model. Another group of isomers of linoleic acid, conjugated linoleic acid (CLA), is present in dairy products and can also be produced* in vivo* by commensal bacteria. Based on competitive scintillation proximity assays, various CLA isomers had IC_50_ values of 3.2–7.4 *μ*M for PPAR*γ*, but had IC_50_ values in the nM range (140–260 nM) for PPAR*α* [26, 48]. This suggests that CLA isomers are stronger activators of PPAR*α* as compared to PPAR*γ*. However, in the past few years there has been a flurry of research investigating the role of CLA isomers in experimental colitis [49] because PPAR*γ* is abundantly expressed in this tissue, and it appears that the protective effects of CLA isomers are due to the activation of PPAR*γ*. Future investigations should consider if these protective effects are being partially mediated by other PPAR isotypes.

#### 2.1.2. Endogenous Lipids

The identification of an endogenous physiological ligand for PPAR*γ* has been problematic, possibly due to its low abundance. Even though it has been well established that endogenous ligand-mediated activation of PPAR*γ* leads to adipocyte differentiation, the identification of this ligand has not yet materialized. Is there any evidence that ligands of PPAR*γ* are generated *in vivo*? Yes, since there are endogenous enzymes that generate lipid ligands that interact with PPARs. 12/15 lipoxygenase-derived oxidized fatty acids such as 13-HODE, 12-HETE, and 15-HETE have been shown to activate PPAR*γ* in vascular smooth muscle cells [50, 51]. In addition, ligands such as 9-HODE, 13-HODE [52], and 1-O-hexadecyl-2-Azelaoyl-sn-glycero-3-phosphocholine (AZ-PC) [24], derived from oxidized LDL, have also been shown to activate PPAR*γ* in cell based studies. Similarly, ligands such as 15-deoxy-Δ,12,14-prostaglandin J2 (PGJ2) generated by the action of cyclooxygenase (COX) on arachidonic acid (n20 : 4) are excellent activators of PPAR*γ* [53] but due to their low *in vivo* abundance are considered as weak ligands.

### 2.2. Dietary Isoflavones

The primary dietary sources of isoflavones that are used as supplements are extracted from legumes, especially soybeans. The isoflavones in soy are mainly daidzein, genistein, and glycitein. After hydrolysis in the gastrointestinal tract, isoflavones are further modified by intestinal microflora. Thus, the metabolites of isoflavones that end up in the circulation depend on the type of microflora that inhabits the intestine. Equol and O-desmethylangolensin (ODMA) are the most common metabolites of daidzein. Several studies have shown that genestein activates PPAR*γ* at micromolar concentration [54, 55] but inhibits adipogenesis in 3T3-L1 adipocytes [56], primary human adipocytes [57], and in animal models [58, 59]. This antiadipogenic effect of genestein is attributed to mechanisms beyond PPAR*γ* activation. For example, downregulation of adipocyte-specific genes such as C/EBP*α* and *β*, PPAR*γ*, SREBP-1, and HSL has been reported [60]. A study by Dang et al. demonstrated that genistein has concentration-dependent effects on progenitor cells, that is, genistein can act as an agonist of the estrogen receptor at lower concentrations (<1 *μ*M) but become a PPAR*γ* agonist at higher concentrations (>1 *μ*M) in mesenchymal progenitor cells, thus promoting either osteogenesis or adipogenesis, respectively [27]. Moreover, a role for the estrogen receptor cannot be overlooked because genistein down regulated ER*α* and ER*β* in an animal study of ovariectomized mice [61]. Daidzein and its metabolite equol activated PPAR*γ* [28] in luciferase reporter assays utilizing several cell types and promoted adipogenesis in 3T3-L1 cells at much lower concentrations (10–100 *μ*m) than genistein [29]. 

### 2.3. Other Dietary Constituents

Fruits and vegetables are rich in flavonoids. By screening a natural product library, Salam and colleagues [30] identified two flavonoids, Ψ-baptigenin (EC_50_ = 2.9 *μ*M) and hesperidin (EC_50_ = 6.6 *μ*M) as strong agonists of PPAR*γ*. Furthermore, these flavonoids promoted a strong induction of PPAR*γ* in THP-1 cells which was abolished by treatment with the PPAR*γ* antagonist GW9662. Interestingly, in a recent study [62], healthy humans who ingested a supplement of Red Clover had detectable levels of Ψ-baptigenin in their plasma, thus making this a plausible physiological ligand of PPAR*γ*. The biological effects of these natural PPAR*γ* agonists need further investigation. Other dietary components that have been studied are epigallocatechin gallate (EGCG, from green tea) and resveratrol (abundant in grapes, wine, and peanuts). Once again, there are very few studies that demonstrate the PPAR*γ* binding ability of these compounds. Because of their ability to reduce lipid accumulation [63] by altering PPAR*γ* expression [64], these agents are presumptive ligands of PPAR*γ*. In an extensive review on culinary herbs and spices, Jungbauer and Medjakovic [31] identified components of herbs and spices such as cinnamon, oregano, and marjoram with PPAR*γ* binding affinities between 2.8 and 23.7 *μ*M. Interestingly, most of these components seem to be very weak transactivators of PPAR*γ*.

In summary, it is obvious that dietary components can bind and activate PPAR gamma. What is lacking, however, is the delineation of the metabolic effects that are specific to this PPAR gamma activation. Thus, future efforts should focus on study methodologies and techniques that can demonstrate a cause and effect relationship between nutraceutical activation of PPAR gamma and its physiological function.

## 3. Toxicology of Nutraceuticals

Nutraceuticals are increasingly being used as nutritional supplements in treatment of diseases. Due to the plant origin of these supplements they are considered safe for human consumption. However, the levels of the active substance consumed vary when taken as a whole food, as compared to a nutritional supplement [65, 66]. Very few studies have reported on long-term effects of nutrition supplements in humans. High consumption of lipids is associated with high risk of cardiovascular disease, diabetes, obesity, and cancer [67, 68]. Higher consumption of flavonoid supplements can alter the physiological levels of iron, vitamins, and other nutrients [66]. Flavonoids also interact with cytochrome P450 enzymes thus altering pharmacodynamics and pharmacokinetics of various drugs [69–71]. Similar to reports on TZDs, some of the flavonoids such as genestein have been associated with increased cancer risk [72–75]. Therefore, unless safety profiles of these nutraceutical supplements in humans are available, caution should be used in their long-term use as PPAR modulators.

## 4. Conclusions

The study of nutraceuticals as PPAR ligands is in its infancy. Newer insights into the role of PPARs in physiology and pathophysiology will help design better therapeutics. Future studies utilizing both high throughput screening technology and tissue specific metabolic profiling should identify nutraceuticals that modulate PPAR*γ* activity. Subsequent cell culture and animal studies followed by rigorous clinical trials should then be able to establish the pharmacological and toxicological profiles of these nutraceuticals and their potential in influencing human health.

## Figures and Tables

**Table 1:** Potential dietary PPAR*γ* ligands.

Ligand	Binding affinity	Type of assay	Reference
Linoleic acid	*K_i_* > 1 *μ*M	Competitive radio-labeled binding assay	[22]
Nitrolinoleic acid	*K_i_* = 133 nM		
9-Hydroxyoctadecadienoic acid (9-HODE)			
13-Hydroxyoctadecadienoic acid (13-HODE)			

9/10-NO_2_-linoleic acid	IC_50_ = 0.6 *μ*M	Scintillation proximity	[23]
12-NO_2_-linoleic acid	IC_50_ = 0.41 *μ*M	Competitive binding assay	
13-NO_2_-linoleic acid	IC_50_ = 0.44 *μ*M		

Azelaoyl phosphatidylcholine (in oxidized LDL)	40 nm	Radiolabeled binding assay	[24]

Docosahexaenoic acid (DHA)	EC_50_ > 10 *μ*m	Dual luciferase reporter system	[25]
4-Hydroxy docosahexaenoic acid (4-HDHA)	EC_50_ = 3.7 *μ*m		
4-Oxodocosahexaenoic acid (4-oxo-DHA)	EC_50_ = 0.4 *μ*M		

Conjugated linoleic acid isomers (CLA)	IC_50_ = 3.2–7.4 *μ*M	Competitive scintillation proximity assays	[26]

Isoflavones:			
Genistein	*K_i_* = 5.7 *μ*M	Membrane-bound competitive PPAR*γ* binding assay	[27]
Daidzein	20 *μ*M	Luciferase reporter assay in 3T3-L1 cells	[28]
	EC_50_ = 73 *μ*M	Luciferase reporter assay in HeLa cells	[29]
Equol	20 *μ*M	Luciferase reporter assay in 3T3-L1 cells	[28]
Biochanin A	EC_50_ = 3.7 *μ*m	Luciferase reporter assay in HeLa cells	[29]
	EC_50_ < 1 *μ*M	Luciferase reporter assay in HepG2 cells	[29]

Flavonoids:			
Psi-baptigenin	EC_50_ = 2.9 *μ*M	Transcriptional factor activity assay in ThP-1 cells	[30]
Hesperidin	EC_50_ = 6.6 *μ*M		

Quercetin (from dill, bay leaves, and oregano)	EC_50_ = 2.8 *μ*M	Ligand screening assay	[31]

2^′^-Hydroxy chalcone (cinnamon in polymeric form)	EC_50_ = 3.8 *μ*M	Ligand screening assay	[31]

Rosmarinic acid (marjoram)	EC_50_ = 16 *μ*M	Ligand screening assay	[31]
